# Dynamic elite strategy mayfly algorithm

**DOI:** 10.1371/journal.pone.0273155

**Published:** 2022-08-25

**Authors:** Qianhang Du, Honghao Zhu

**Affiliations:** School of Computer and Information Engineering, Bengbu University, Bengbu, Anhui 233030, China; UCSI University, MALAYSIA

## Abstract

The mayfly algorithm (MA), as a newly proposed intelligent optimization algorithm, is found that easy to fall into the local optimum and slow convergence speed. To address this, an improved mayfly algorithm based on dynamic elite strategy (DESMA) is proposed in this paper. Specifically, it first determines the specific space near the best mayfly in the current population, and dynamically sets the search radius. Then generating a certain number of elite mayflies within this range. Finally, the best one among the newly generated elite mayflies is selected to replace the best mayfly in the current population when the fitness value of elite mayfly is better than that of the best mayfly. Experimental results on 28 standard benchmark test functions from CEC2013 show that our proposed algorithm outperforms its peers in terms of accuracy speed and stability.

## 1. Introduction

With the development of technology, there are a large number of optimization problems in real life, and such problems usually have the characteristics of nonlinearity and high dimensionality [1]. In the early, traditional methods, such as the Newton method [2] and gradient descent method [3], are considered an effective ways to solve these problems, they can obtain the results within a reasonable time. However, the traditional methods have the following limits: a) they are suitable for dealing with small-scale problems, and b) they require that the problems must be differentiable. Thus, they are not the best choice when problems become more and more complex. It is found that swarm intelligence optimization algorithms, which are inspired by the behavior of natural biological groups, are suitable for solving large-scale problems, and thus they have attracted the attention of more researchers. Up to now, more and more swarm intelligence optimization algorithms have been proposed to solve various optimization problems, such as Particle Swarm Optimization (PSO) [4], Grey Wolf Optimization (GWO) [5], Artificial Bee Colony Algorithm (ABC) [6], Harris Hawks Optimization (HHO) [7], Symbiotic Organisms Search Algorithm (SOS) [8], Firework Algorithm (FWA) [9], Monarch Butterfly Optimization (MBO) [10], Slime Mould Algorithm (SMA) [11], Moth Search Algorithm (MSA) [12], Hunger Games Search (HGS) [13], Runge Kutta Method (RUN) [14], Colony Predation Algorithm (CPA) [15], and Weighted Mean of Vectors (INFO) [16], etc. They have been widely used in various fields [17–21].

In addition, Li, etc. [22] proposes a novel PSO algorithm that converges on the global optimal solution quickly and accurately for dynamic adjustment controller parameters. Zhao, etc. [23] proposes an MSCEWT (enhanced empirical wavelet transform) based on the maximum-minimum length curve method to realize fault diagnosis of motor bearings. Ran, etc. [24] proposes an improved K-means clustering algorithm based on a noise algorithm to capture urban hotspots. Wu, etc. [25] proposes a hybrid optimization algorithm combining computational intelligence techniques to solve the multifactor highway passenger volume prediction problem. Liu, etc. [26] designed an MVDE (Mixed-Variable Differentiate Evolution) as the scheduling algorithm to adapt to the new problem model. Zhou, etc. [27] proposes a self-adaptive differential evolution algorithm to assign jobs into batches without breaking the machine capacity constraint and then sort the batches to minimize the makespan. Zhao, etc. [28] proposes an SD-Jaya (self-learning discrete Jaya algorithm) to address the energy-efficient distributed no-idle flow-shop scheduling problem (FSP) in a heterogeneous factory system. Zhao, etc. [29] proposes a two-stage cooperative evolutionary algorithm with problem-specific knowledge called TS-CEA to address energy-efficient scheduling of the no-wait flow-shop problem (EENWFSP) with the criteria of minimizing both makespan and total energy consumption. And, Zhao, etc. [30] proposes an ensemble discrete differential evolution (EDE) algorithm to solve the blocking flow-shop scheduling problem. Based on the free lunch theorem [31], even though these algorithms have achieved certain results on some problems, there are still some shortcomings such as low solution accuracy, slow convergence speed, and easy falling into local optimum for some other problems.

Mayfly Algorithm (MA) [32] was proposed as a new swarm intelligence optimization algorithm in 2020. It combines the advantages of multiple classical optimization algorithms such as PSO [4], genetic algorithm (GA) [33] and firefly algorithm (FA) [34]. MA and its variants have been used in various industries, such as feature selection [35], Industrial optimization [36], ensemble forecasting system [37], photovoltaic systems [38].

MA is inspired by the actual behavior of mayflies, i.e., the attraction of males to females. It follows the principles of crossover, mutation and selection [39]. MA has better convergence speed and convergence accuracy than other swarm intelligence optimization algorithms when solving the optimization problems. Like other algorithms, it is also easy to fall into a local optimum. Moreover, the search space will also increase exponentially due to the increase of the dimension of problems, leading to failure to achieve the expected result when paying a lot of time cost.

Therefore, in order to effectively help the MA to jump out of a local optimum, an improved mayfly algorithm based on dynamic elite strategy (DESMA) is proposed. Specifically, in each iteration, the best mayfly is first selected in current population, and then the dynamic elite strategy is applied to it. In the proposed dynamic elite strategy, the search radius of the selected one is dynamically adjusted based on whether to find a better solution. Also, a number of elite mayflies are generated around the search radius. Finally, if the newly generated elite mayflies are better than the best one among the current population, the best one is replaced with the newly generated elite mayfly.

In summary, the novelty and main contributions of this article are summarized as follows:

1. In order to improve the performance of the basic MA, an improved mayfly algorithm based on dynamic elite strategy (DESMA) is proposed in this work. In it, the search radius is dynamically set, and a certain number of elite mayflies are generated within this range.
2. According to the elite selection strategy, the dynamic search range can effectively help jump out of the local optimum, and the elite mayfly with the best fitness value is selected to replace the current global mayfly when its fitness value is better than that of the global one. It improves the global search ability of the algorithm, thereby helping the algorithm to achieve a better result.
3. In order to evaluate the performance of the proposed algorithm, 28 standard benchmark test functions are selected for simulation [40–45]. The experimental results show that the DESMA algorithm has better improvement in terms of solution accuracy and speed, It is also significantly better than other comparison algorithms in terms of solution stability.

The remainder of this article is organized as follows. The basic MA is introduced in Section II. The proposed algorithm is presented and discussed in Section III. Experimental results are given in Section IV. Section V concludes this article.

## 2. Mayfly algorithm

MA is inspired by the social behavior of mayflies. Most adult male mayflies usually gather on the water surface and attract female mayflies to complete reproduction through the unique wedding dance between mayflies. At this time, male mayflies are affected by the population and the position of themselves, and they can constantly approach the position of the optimal solution. Among the progeny mayflies produced by mating, there will be very few mutation mayflies, and they will continue to participate in the optimization with the parent mayflies. After that, all individuals in the population will use the elite retention strategy to achieve a new population for the next iteration.

The mayfly algorithm initially randomly generates two populations of mayflies, namely male and female mayflies, and each mayfly in the two populations is randomly generated in the search space. The position of the mayfly is represented by a *D*-dimensional vector *x*_*i*_ = {*x*_1_, *x*_2_, *x*_3_,…,*x*_*D*_}, and the speed is represented as *V*_*i*_ = {*V*_1_, *V*_2_, *V*_3_,…,*V*_*D*_}, In each iteration, the mayfly will move towards the local best position (pbest) and the global best position (gbest).

### 2.1. Movement of male mayflies

Male mayflies gather together, and the male mayflies make corresponding position adjustments according to their location and the location information of the population. Suppose $x_{i}^{t}$ is the position of the *i*-th male mayfly *x*_*i*_ at the generation *t* in the search space, and add the velocity $v_{i}^{t+1}$ to the current position to change its position, the formula is as follows:

$$x_{i}^{t+1}=x_{i}^{t}+v_{i}^{t+1}$$ (1)

The Cartesian distance formula is:

$$\|x_{i}-X_{i}\|=\sqrt{\sum_{j=1}^{n}{\left(x_{ij}-X_{ij}\right)}^{2}}$$ (2)

where *x*_*ij*_ represents the position of the mayfly *i* in the *j* dimension, and *X*_*ij*_ represents the value of pbest or gbest in the *j* dimension. In addition, male mayflies always attract female mayflies through wedding dances on the water. Assuming that male mayflies cannot move quickly, the formula for calculating the speed of male mayflies is:

$$v_{ij}^{t+1}=\left\{ \begin{array}{c} g*v_{ij}^{t}+a_{1}e^{-\beta r_{p}^{2}}({pbest}_{ij}-x_{ij}^{t})+a_{2}e^{-\beta r_{g}^{2}}({gbest}_{j}-x_{ij}^{t}),\;if\;f(gbest)>f(x_{i})\\ g*v_{ij}^{t}+d*r,\;if\;f(gbest)\leq f(x_{i}) \end{array}\right.$$ (3)

where $v_{ij}^{t+1}$ represents the speed of the *i*-th mayfly *i* at the generation *t*+1 in the *j* dimension, $x_{i}^{t}$ represents the position of the mayfly *x*_*i*_ at the generation *t* in the search space, *a*_1_ is the population learning coefficient, *a*_2_ is the individual learning coefficient, *β* is the visibility coefficient, *g* is the dynamic inertia weight, *d* is the dance coefficient; *r*_*p*_ represents the Cartesian distance between the current position and *pbest*_*ij*_, *r*_*g*_ represents the Cartesian distance between the current position and *gbest*_*j*_; *r* is a random number following [−1,1].

### 2.2. Movement of female mayflies

Unlike male mayflies, female mayflies do not gather together in groups, and female mayflies will actively approach male mayflies with better fitness and reproduce. Suppose $y_{i}^{t}$ is the position of the *i*-th female mayfly *y*_*i*_ at the generation *t* in the search space, and add the velocity $v_{i}^{t+1}$ to the current position to change the position, the formula is as follows:

$$y_{i}^{t+1}=y_{i}^{t}+v_{i}^{t+1}$$ (4)

In addition, the mutual attraction of male and female mayflies is a deterministic process. In it, the optimal female mayflies will be attracted by the optimal male mayflies, the second-best female mayflies will be attracted by the second-best male mayflies, and so on. If the fitness of the male mayfly is poor, the corresponding female mayfly will randomly fly around. Then the formula for calculating the speed of the female mayfly is:

$$v_{ij}^{t+1}=\left\{ \begin{array}{c} g*v_{ij}^{t}+a_{2}e^{-\beta r_{mf}^{2}}(x_{ij}-y_{ij}^{t}),\;if\;f(y_{i})>f(x_{i})\\ g*v_{ij}^{t}+f_{l}*r,\;if\;f(y_{i})\leq f(x_{i}) \end{array}\right.$$ (5)

where *r*_*mf*_ is the Cartesian distance between female and male mayflies, and *f*_*l*_ is the random flight coefficient between [−1,1].

### 2.3. Mayfly mating

In MA, male and female mayflies are selected for mating to produce offspring. The mating result includes two offspring, and the formula is as follows:

offs1=L*male+(1−L)*female (6)

offs2=L*female+(1−L)*male (7)

Where *offs*1 *and offs*2 are two offspring, *male* is a male mayfly, *female* is a female mayfly, and *L* is a random number between [−1,1].

### 2.4. Gaussian variation

To deal with the precocious phenomenon, MA introduces Gaussian mutation aiming to jump out of the local optimum. The progeny mayfly with the mutation will appear randomly in any dimension. The formula is as follows:

$${offs}_{n}={offs}_{n}+\sigma N_{n}(0,1)$$ (8)

where *n* is the dimension of the offspring mayfly, *σ* is the standard deviation of Gaussian variation, *N*_*n*_(0,1) is the standard normal distribution with mean 0 and variance 1.

### 2.5. Dynamic inertia

To balance local and global search ability and find the global optimal solution as quickly as possible, the MA algorithm introduces dynamic inertia weights. The formula is as follows:

$$g=g_{max}-\frac{g_{max}-g_{min}}{{iter}_{max}}*iter$$ (9)

where *g* is the inertia weight, *g*_*max*_ is the maximum inertia weight, *g*_*min*_ is the minimum inertia weight, *iter* is the number of iterations, and *iter*_*max*_ is the maximum number of iterations.

### 2.6. Wedding dance factor and random flight factor

In order to find the global optimal solution faster and more accurately, the wedding dance coefficient and random flight coefficient will increase the local search ability with the number of iterations, thereby improving the convergence accuracy. The formula is as follows:

$$d^{t}=d^{1}*{\delta_{1}}^{t},0<\delta_{1}<1$$ (10)

$${f_{l}}^{t}={f_{l}}^{1}*{\delta_{2}}^{t},0<\delta_{2}<1$$ (11)

where *d*^*t*^ and *f*_*l*_^*t*^ are the wedding dance coefficient and random flight coefficient at time *t*, and δ is the attenuation parameter.

### 2.7. The frame of mayfly algorithm

To summarize the whole process of MA the pseudocode is added, the details are shown in Algorithm 1 [32].

Algorithm 1 Mayfly algorithm

Input: *x*_*ij*_, *X*_*ij*_

Output: Best location

1: While stopping criteria are not met

2: Update velocities and solutions of males and females

3: Evaluate solutions

4: Rand the mayflies

5: Mate the mayflies

6: Evaluate offspring

7: Separate offspring to male and female randomly

8: Replace the worst solutions with the best new ones

9: Update pbest and gbest

10: End

## 3. Mayfly algorithm for dynamic elite strategy

MA is a new type of swarm intelligence optimization algorithm, which is still in the initial stage of research. From the content point of view, the algorithm process is simple and easy to understand; from the operation effect point of view, the algorithm has faster convergence speed and better convergence accuracy than other algorithms. Based on the social nature of the mayfly population, MA classifies the mayfly population into male and female mayfly populations. When the individual population moves, the male mayfly acts as the movement condition of the female mayfly, while the male mayfly takes the global optimal position as the basis for movement. In the search space, the positions of mayflies in the initial stage are randomly distributed. In each iteration, the optimal male mayfly individual in the population will be compared with the global optimal individual, and then a new global optimal solution will be obtained. However, in the later stage of the algorithm, most of the optimal male mayfly individuals will be concentrated near the global optimal solution, thereby falling into the local optimal solution.

In order to solve the above problems, this paper proposes an improved Mayfly Algorithm Based on Dynamic Elite Strategy (DESMA), which starts from the global optimal solution and performs a more accurate elite selection strategy near the global optimal solution. On the one hand, the algorithm can jump out of the local optimum, improve the diversity of the population, expand the search range, and possibly find a new global optimal solution that is better than the optimal global one of the previous generation; on the other hand, in the case of ensuring the integrity of the population, it not only improves the convergence speed, but also improves the convergence accuracy, and can find the optimal global solution more stably. Next, it is introduced in detail.

### 3.1. Dynamic elite strategy

After each iteration, the DESMA will compare the current global optimal solution with the global optimal one of the previous generations, and then determine a specific space and its search range near the position of the new global optimal solution. If the fitness value of the current optimal solution is better than that of the global optimal one in the previous generation, the search range is expanded, and the global optimal solution is the current global optimal solution; otherwise, the search range is narrowed, and the current global optimal solution is still the global optimal solution of the previous generation. Based on the above analysis, for a minimization problem, the fitness value of the current global optimal solution will always remain less than or equal to the fitness value of the previous generation’s global optimal solution. When the mayfly finds the current global optimal solution position, it will determine the search range of a specific space around the mayfly. The formula is as follows:

$$R=\left\{ \begin{array}{c} R*c_{1},if\;f(cgbest)<f(lgbest)\\ R*c_{2},if\;f(cgbest)\geq f(lgbest) \end{array}\right.$$ (12)

where *R* is the search range in a specific space, *c*_1_ is the enlargement factor, it is set to a constant 1.05, *c*_2_ is the reduction factor, it is set to a constant 0.95, *cgbest* is the current global optimal position, and *lgbest* is the previous generation global optimal position.

It can be seen from Formula (12) that if this generation finds a better solution than the previous generation, it means that the current region has great potential, and thus the search range is enlarged aiming to ensure that the region can be fully searched. On the contrary, if this generation does not find a better solution, it means that the current area is poor, so the search range is narrowed down so that a finer search can be done in a smaller range.

After determining the search range of a specific space and the position of the new global optimal solution, a number in n-dimension is randomly generated to prepare for generating the elite mayflies. The formula is as follows:

$$r_{1}=2*rand(1,n)-1$$ (13)

where *r*_1_ is a random number between [−1,1], and *n* is the dimension.

After that, a dynamic search is performed in a specific space to generate the positions of *k* elite mayflies. The formula is as follows:

$$egbest=cgbest+r_{1}*R$$ (14)

Where *egbest* is the elite mayfly generated within the search range, *cgbest* is the current global optimal solution; *r*_1_ is a random number between [−1,1]; *R* is the initially determined search range.

In order to avoid exceeding the search range when searching for the elite mayfly, thereby causing the wrong mayfly to affect the global optimal solution of the population, the upper and lower bounds of the *n*-dimensional search space are set, and the formula is as follows:

$$\left\{ \begin{array}{c} egbest=\max\;\left(egbest,LowerBound\right)\\ egbest=min\;(egbest,UpperBound) \end{array}\right.$$ (15)

where *LowerBound* is the lower bound of the search space, and *UpperBound* is the upper bound of the search space.

Determining the position of the elite mayfly within the range of the new global optimal solution, and replacing the position of the globally optimal solution obtained directly with the position of the elite mayfly generated by the dynamic elite strategy. The modified speed formula is as follows:

$$v_{ij}^{t+1}=\left\{ \begin{array}{c} g*v_{ij}^{t}+a_{1}e^{-\beta r_{p}^{2}}({pbest}_{ij}-x_{ij}^{t})+a_{2}e^{-\beta r_{g}^{2}}({egbest}_{j}-x_{ij}^{t}),if\;f(egbest)>f(x_{i})\\ g*v_{ij}^{t}+d*r,if\;f(egbest)\leq f(x_{i}) \end{array}\right.$$ (16)

### 3.2. The process of DESMA

(1) The complete framework of DESMA is shown in Algorithm 2.

Algorithm 2 DESMA

Input: *x*_*ij*_, *X*_*ij*_ // Initialize parameters, the position and velocity of male and female mayflies, calculate and find the current global optimal solution position.

Output: Best location

1: Update the speed and position of male and female mayflies according to Formulas (1) (2) (3) (4) (5).

2: Sort according to the current mayfly fitness value

3: Generate progeny mayflies according to Formulas (6), (7), and randomly generate mutant offspring according to Gaussian mutation Formula (8).

4: According to the fitness value of the population, sort the male and female mayflies, and replace the inferior solution with the current better solution.

5: Improve the local search ability and convergence accuracy according to Formulas (10) and (11), and balance the global exploration ability and local search ability according to Formula (9).

6: Determine the search range in a specific space according to Formula (12), and then generate a random number between [–1, 1] by Formula (13), find *k* elite mayflies, and determine the best elite mayflies.

7: In order to prevent the elite mayfly from exceeding the search range when searching, the upper and lower bounds of the search are determined according to Formulas (14) and (15).

8: Determine whether the maximum iteration is reached. If yes, go to Step 11. If not, go to Step 3.

9: Bring the position of the elite mayfly into Formula (16), and output the global optimal solution obtained by the elite mayfly.

10:End

**Fig 1:**
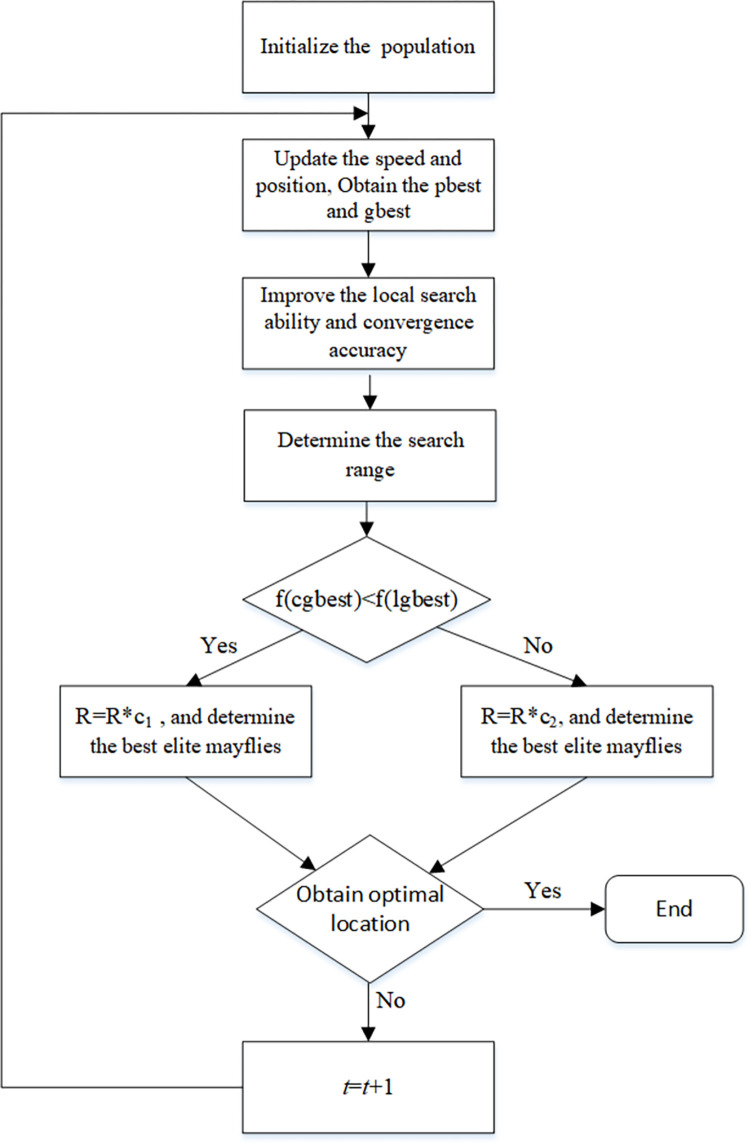
Flow chart of the DESMA.

(2) In order to more clearly express DESMA, which the flow chart is given in Fig 1.

### 3.3. Time complexity analysis

Usually, the time complexity of an algorithm is related to the specific operations such as addition, subtraction, multiplication, and division of the algorithm [46]. Assuming that the number of mayflies in the DESMA algorithm is *N*, the number of progeny mayflies is *M*, and the number of elite mayflies is *k*, the time complexity is analyzed according to the execution steps of the algorithm.

1. The number of executions for initializing various parameters is 1 time, so the time complexity of step (1) is *O(1)*.
2. The mayfly population needs to perform *N* operations for randomly initializing the initial positions and velocities of male and female mayflies. Therefore, the time complexity of step (2) is *O(N+N)*.
3. Update the speed and position of the male and female mayflies, and the number of executions also requires *N* times each. Therefore, the time complexity of step (3) is *O(N+N)*.
4. The number of executions sorted according to the current mayfly fitness value is *2Nlg2N*, therefore, the time complexity of step (4) is *O(2Nlg2N)*.
5. According to the Gaussian mutation formula, the mutant offspring are randomly generated and executed *M/2* times. Therefore, the time complexity of step (5) is *O(M/2)*.
6. Sort the male and female mayflies again, and replace the inferior solution with the current better solution. The number of executions is *2Nlg2N* Therefore, the time complexity of step (6) is *O(2Nlg2N)*.
7. Balancing the global exploration ability and the local search ability is performed for *N* times, so the time complexity of step (7) is *O(N)*.
8. According to the determined search range in a specific space, *k* elite mayflies are found, and the number of executions is *k* times. Therefore, the time complexity of step (8) is *O(k)*.
9. In order to prevent the elite mayfly from searching beyond the search range, the number of executions is also *k* times. Therefore, the time complexity of step (9) is *O(k)*.
10. The number of executions to determine whether the upper limit of iteration is reached is *N* times, so the time complexity of step (10) is O(N).
11. The position of the elite mayfly is brought into the relevant formula, and the number of executions is 1. Therefore, the time complexity of step (11) is *O(1)*.

After the above steps, the time complexity of the DESMA algorithm after NC iterations is *O(NC×(6N+M/2+4Nlg2N+2k+2))*.

## 4. Simulation experiment and analysis

### 4.1. Test function and parameter settings

To test the performance of the proposed DESMA, the 28 international standard Benchmark test functions are selected in this work, which is listed in Table 1 [40], where Function type is the function type, the Function number is the function sequence, Function name is the function name, Range is the value range, and Optimal value is the optimal value. According to its properties, it can be divided into unimodal functions (*f1~f5*), multimodal functions (*f6~f20*), and mixed functions (*f21~f28*). The experimental environment is Intel i7, RAM 8.0GB, Windows10 operating system, MATLAB R2018a. In the experiment, each function is repeated 51 times, the dimension is set to 30, and the maximum number of evaluation times is 300,000. At the same time, the average error and average ranking [47] are used as evaluation measures, and the average error in this paper is represented by $\bar{e}$, and the formula as follows:

$$\bar{e}=\frac{\sum_{i=1}^{m}\left|f_{i}(x)-f_{o}(x)\right|}{m}$$ (17)

where *f*_*i*_(*x*) is the actual value calculated; *f*_*o*_(*x*) is the optimal value, and *m* is the number of times each function runs. The average rank is represented by *R*^*α*^ with the following formula:

$$R^{\alpha }=\frac{\sum_{j=1}^{r}R_{j}^{\alpha }}{r}$$ (18)

where *α* is a hyperparameter or method; $R_{j}^{\alpha }$ is the role played in the experiment by the number *k* of elite mayflies generated when the *j*-th function is optimized, *r* is the total number of functions participating in the experiment.

**Table 1:** Standard benchmark 28 test functions.

Function type	Function number	Function name	Ranges	Optimal value
Unimodal Function	*f1*	Sphere function	[–100,100]	-1400
	*f2*	Rotated high conditioned elliptic	[–100,100]	-1300
	*f3*	Rotated bent cigar function	[–100,100]	-1200
	*f4*	Rotated discus function	[–100,100]	-1100
	*f5*	Different powers function	[–100,100]	-1000
Basic Multimodal Function	*f6*	Rotated rosenbrock’s function	[–100,100]	-900
	*f7*	Rotated schaffers F7 function	[–100,100]	-800
	*f8*	Rotated Ackley’s function	[–100,100]	-700
	*f9*	Rotated weierstrass function	[–100,100]	-600
	*f10*	Rotated griewank’s function	[–100,100]	-500
	*f11*	Rastrigin’s function	[–100,100]	-400
	*f12*	Rotated rastrigin’s function	[–100,100]	-300
	*f13*	Non-continuous rotated rastrigin’s function	[–100,100]	-200
	*f14*	Schewefel’s function	[–100,100]	-100
	*f15*	Rotated schewefel’s function	[–100,100]	100
	*f16*	Rotated kstsuura function	[–100,100]	200
	*f17*	Lunacek Bi_Rastrigin function	[–100,100]	300
	*f18*	Rotated lunacek Bi_Rastrigin function	[–100,100]	400
	*f19*	Expanded griewank’s plus Rosenbrock’s function	[–100,100]	500
	*f20*	Expanded scaffer’s F6 function	[–100,100]	600
Composition Funtion	*f21*	Composition function 1 (N = 5)	[–100,100]	700
	*f22*	Composition function 2 (N = 3)	[–100,100]	800
	*f23*	Composition function 3 (N = 3)	[–100,100]	900
	*f24*	Composition function 4 (N = 3)	[–100,100]	1000
	*f25*	Composition function 5 (N = 3)	[–100,100]	1100
	*f26*	Composition function 6 (N = 5)	[–100,100]	1200
	*f27*	Composition function 7 (N = 5)	[–100,100]	1300
	*f28*	Composition function 8 (N = 5)	[–100,100]	1400

### 4.2. Sensitivity analysis of elite selection strategies

To evaluate the effect of setting parameters of DESMA on the convergence progress and speed. This paper discusses and analyzes the generation number *k* of elite mayflies within the search range in the elite mayfly selection strategy, and then determines the optimal number *k* of elite mayflies. For a given value of *k*, if the average ranking obtained is the smallest, the value of *k* is better than other values. In this section, the number *k* is set to 5, 10, 15, 20, and 25, respectively. The q stands for rank in the same set of experiments. The results are presented in Table 2 when *k* takes different values.

**Table 2:** Comparison of the results of different elite mayfly numbers.

Functions	*k* = 5	*k* = 10	*k* = 15	*k* = 20	*k* = 25
	$\bar{e}/q$	$\bar{e}/q$	$\bar{e}/q$	$\bar{e}/q$	$\bar{e}/q$
*f1*	**0.00E+00/1**	**0.00E+00/1**	**0.00E+00/1**	**0.00E+00/1**	**0.00E+00/1**
*f2*	**5.29E+05/1**	7.41E+05/2	9.22E+05/3	1.47E+06/4	1.51E+06/5
*f3*	**6.23E+08/1**	6.73E+08/2	6.99E+08/3	8.43E+08/4	1.20E+09/5
*f4*	**6.02E+04/1**	6.06E+04/2	7.25E+04/5	6.41E+04/3	7.21E+04/4
*f5*	**0.00E+00/1**	**0.00E+00/1**	**0.00E+00/1**	**0.00E+00/1**	**0.00E+00/1**
*f6*	**2.51E+01/1**	2.58E+01/2	3.67E+01/5	3.05E+01/3	3.33E+01/4
*f7*	1.33E+02/5	1.27E+02/2	**1.21E+02/1**	1.29E+02/3	1.32E+02/4
*f8*	**2.10E+01/1**	**2.10E+01/1**	**2.10E+01/1**	**2.10E+01/1**	**2.10E+01/1**
*f9*	2.99E+01/5	2.94E+01/2	**2.90E+01/1**	2.96E+01/3	2.97E+01/4
*f10*	1.71E-01/5	1.40E-01/3	**1.31E-01/1**	1.50E-01/4	1.36E-01/2
*f11*	**2.43E-01/1**	5.66E-01/5	4.29E-01/3	4.22E-01/2	5.07E-01/4
*f12*	1.68E+02/3	**1.12E+02/1**	1.83E+02/5	1.73E+02/4	1.60E+02/2
*f13*	2.30E+02/5	2.17E+02/2	2.19E+02/3	2.24E+02/4	**2.10E+02/1**
*f14*	9.16E+02/4	9.37E+02/5	9.12E+02/3	**8.65E+02/1**	9.06E+02/2
*f15*	4.46E+03/4	4.01E+03/2	4.47E+03/5	4.35E+03/3	**3.99E+03/1**
*f16*	1.67E+00/5	1.36E+00/4	**9.29E-01/1**	9.76E-01/3	9.65E-01/2
*f17*	**3.36E+01/1**	3.39E+01/2	3.45E+01/3	3.47E+01/4	3.54E+01/5
*f18*	1.66E+02/5	1.44E+02/4	**1.32E+02/1**	1.37E+02/3	1.36E+02/2
*f19*	3.26E+00/4	3.16E+00/3	3.70E+00/5	3.09E+00/2	**3.03E+00/1**
*f20*	1.32E+01/4	**1.29E+01/1**	1.32E+01/4	1.30E+01/2	1.31E+01/3
*f21*	2.98E+02/5	**2.75E+02/1**	2.97E+02/4	2.95E+02/3	2.89E+02/2
*f22*	6.99E+02/3	6.86E+02/2	**6.78E+02/1**	7.14E+02/4	7.84E+02/5
*f23*	4.95E+03/5	**4.11E+03/1**	4.64E+03/3	4.56E+03/2	4.68E+03/4
*f24*	2.86E+02/4	2.84E+02/3	2.86E+02/4	**2.80E+02/1**	2.81E+02/2
*f25*	2.97E+02/5	**2.66E+02/1**	2.91E+02/2	2.95E+02/4	2.92E+02/3
*f26*	3.14E+02/5	3.10E+02/4	**3.01E+02/1**	3.03E+02/2	3.04E+02/3
*f27*	1.09E+03/3	1.09E+03/3	1.07E+03/2	**1.03E+03/1**	1.07E+03/2
*f28*	5.23E+02/5	**4.01E+02/1**	4.75E+02/3	4.32E+02/2	5.01E+02/4
*R* ^*α*^	3.32	**2.25**	2.68	2.64	2.82

From the simulation results in Table 2, it can be seen that when the number of elite mayflies *k* = 5, the DESMA has a more accurate optimization ability in the unimodal function compared with other *k*’s, while it has poor performance on the other functions, and thus the final average ranking is the worst. When *k* = 15, the DESMA can find more optimal solutions on some multimodal functions and mixed functions, but it has no outstanding performance on unimodal functions. When *k* = 20 and *k* = 25, even though the DESMA can also find the optimal solution on a few functions, the results on other functions are always poor. Compared with the number of elite mayflies in other settings, the average ranking of *k* = 10 takes first place, followed by *k* = 15 and 20. Therefore, when the number of elite mayflies is set to 10, the DESMA has the best results and the strongest stability.

### 4.3. Algorithm-related parameter settings and algorithm comparison analysis

In this section, the proposed algorithm is compared with the basic Mayfly Algorithm (MA), the Harris Hawks Optimization Algorithm (HHO) [48], the Improved Symbiotic Search Algorithm (ISOS) [49], and the Enhanced Fireworks Algorithm (EFWA) [50], Gradient-Based Optimizer (GBO) [51], Grey Wolf Optimizer (GWO) [5] and Slime Mould Algorithm (SMA) [11]. Each base test function was run 51 times independently, and the mean error and mean execution time were recorded. Other parameters are set as follows: population size *size* = 50; maximum inertia weight *g*_*max*_ = 0.9, minimum inertia weight *g*_*min*_ = 0.4; population learning coefficient *a*_1_ = 1.0, mayfly individual learning coefficient *a*_2_ = 1.5, wedding dance coefficient *d*^*t*^ = 5, The attenuation coefficient of the wedding dance coefficient δ_1_ = 0.8, the random flight coefficient *f*_*l*_^*t*^ = 1, and the attenuation coefficient of the random flight coefficient δ_2_ = 0.99. The specific parameter settings of other algorithms are the same as the original literature [48–50], and the experimental results are shown in Table 3.

**Table 3:** Algorithm test results.

Functions	MA	ISOS	EFWA	HHO	GBO	GWO	SMA	DESMA
	$\bar{e}/q$	$\bar{e}/q$	$\bar{e}/q$	$\bar{e}/q$	$\bar{e}/q$	$\bar{e}/q$	$\bar{e}/q$	$\bar{e}/q$
*f1*	**0.00E+00/1**	**0.00E+00/1**	7.82E-02/3	5.73E+00/4	**0.00E+00/1**	1.92E+03/5	8.31E-04/2	**0.00E+00/1**
*f2*	7.39E+05/3	3.45E+06/6	5.09E+05/2	9.59E+06/7	**1.74E+05/1**	2.13E+07/8	3.10E+06/5	7.41E+05/4
*f3*	1.41E+09/5	1.55E+09/6	2.52E+08/2	1.67E+09/7	**2.46E+08/1**	7.04E+09/8	5.84E+08/3	6.73E+08/4
*f4*	6.11E+04/7	3.61E+01/3	**1.09E+00/1**	7.09E+03/5	1.50E+01/2	314E+04/8	5.70E+01/4	6.06E+04/6
*f5*	**0.00E+00/1**	**0.00E+00/1**	7.85E-02/3	2.23E+00/4	**0.00E+00/1**	1.04E+03/5	7.54E-03/2	**0.00E+00/1**
*f6*	2.76E+01/4	4.73E+01/6	3.39E+01/5	6.36E+01/7	**2.29E+01/1**	1.54E+02/8	2.73E+01/3	2.58E+01/2
*f7*	1.32E+02/7	9.69E+01/2	1.28E+02/6	2.41E+03/8	1.22E+02/4	**5.67E+01/1**	1.02E+02/3	1.27E+02/5
*f8*	2.10E+01/2	2.10E+01/2	2.10E+01/2	**2.09E+01/1**	**2.09E+01/1**	**2.09E+01/1**	**2.09E+01/1**	2.10E+01/2
*f9*	2.97E+01/5	2.63E+01/3	3.19E+01/7	3.63E+01/8	3.06E+01/6	**1.87E+01/1**	2.16E+01/2	2.94E+01/4
*f10*	1.06E-01/2	6.84E-01/5	8.31E-01/6	5.87E+00/7	**1.01E-01/1**	3.93E+02/8	4.35E-01/4	1.40E-01/3
*f11*	**1.56E-01/1**	9.84E+01/4	4.26E+02/8	1.76E+02/7	1.29E+02/6	1.08E+02/5	1.02E+01/3	5.66E-01/2
*f12*	1.71E+02/5	1.69E+02/4	6.13E+02/8	5.72E+02/7	2.13E+02/6	1.38E+02/3	1.19E+02/2	**1.12E+02/1**
*f13*	2.41E+02/5	2.37E+02/4	4.48E+02/7	5.95E+02/8	2.65E+02/6	**1.91E+02/1**	2.04E+02/2	2.17E+02/3
*f14*	**8.58E+02/1**	1.34E+03/4	4.15E+03/8	2.87E+03/6	3.21E+03/7	2.74E+03/5	1.30E+03/3	9.37E+02/2
*f15*	4.81E+03/8	4.24E+03/5	4.40E+03/6	4.73E+03/7	4.23E+03/4	**3.11E+03/1**	4.02E+03/3	4.01E+03/2
*f16*	2.61E+00/8	1.49E+00/5	**6.07E-01/1**	1.71E+00/6	8.56E-01/3	2.47E+00/7	7.76E-01/2	1.36E+00/4
*f17*	**3.37E+01/1**	1.48E+02/4	3.15E+02/7	6.96E+02/8	1.65E+02/5	1.85E+02/6	5.07E+01/3	3.39E+01/2
*f18*	2.02E+02/5	1.98E+02/4	1.75E+02/3	7.28E+02/7	1.69E+02/2	2.68E+02/6	**1.44E+02/1**	**1.44E+02/1**
*f19*	2.95E+00/2	2.86E+01/6	1.09E+01/4	3.59E+01/7	1.26 E+01/5	2.30E+02/8	**2.85E+00/1**	3.16E+00/3
*f20*	1.30E+01/3	**1.26E+01/1**	1.46E+01/5	1.49E+01/6	1.31E+01/4	**1.26E+01/1**	1.50E+01/7	1.29E+01/2
*f21*	3.03E+02/2	3.38E+02/6	3.11E+02/3	3.68E+02/7	3.37E+02/5	1.08E+03/8	3.19E+02/4	**2.75E+02/1**
*f22*	6.99E+02/2	1.59E+03/4	5.46E+03/8	3.44E+03/7	3.04E+03/5	3.17E+03/6	1.27E+03/3	**6.86E+02/1**
*f23*	4.95E+03/5	5.25E+03/6	5.61E+03/7	6.61E+03/8	4.84E+03/4	4.25E+03/3	4.12E+03/2	**4.11E+03/1**
*f24*	2.85E+02/5	2.74E+02/3	3.29E+02/8	3.27E+02/7	2.95E+02/6	**2.49E+02/1**	2.68E+02/2	2.84E+02/4
*f25*	2.96E+02/4	2.91E+02/3	3.55E+02/7	3.39E+02/6	3.08E+02/5	2.75E+02/2	2.75E+02/2	**2.66E+02/1**
*f26*	3.35E+02/6	2.68E+02/2	3.54E+02/7	3.64E+02/8	**2.00E+02/1**	3.13E+02/5	2.82E+02/3	3.10E+02/4
*f27*	1.09E+03/3	9.51E+02/2	1.28E+03/5	1.37E+03/6	1.15E+03/4	9.51E+02/2	**9.04E+02/1**	1.09E+03/3
*f28*	4.50E+02/4	7.72E+02/5	4.76E+03/8	4.53E+03/7	1.27E+03/6	**1.35E+02/1**	3.00E+02/2	4.01E+02/3
*R* ^*α*^	3.82	3.82	5.25	6.54	3.68	4.43	2.68	**2.57**
*t*-test(+, =, -)	15/6/7	15/5/8	18/3/7	19/3/6	16/6/6	17/3/8	13/6/9	**0/0/0**

The experimental results in Table 3 show that the proposed DESMA has achieved nine optimal results, 16-second optimal results, and is better than its peers on most functions, and thus it ranks 1^st^ in terms of *R*^*a*^. The original MA algorithm performs better on six functions. The DESMA algorithm has more advantages than other algorithms in the mixed functions *f21~f28*, and the convergence accuracy is higher, but the optimization ability of the unimodal function *f1~f5* is slightly inferior to the EFWA algorithm. In MA and ISOS algorithms, most of them only achieve suboptimal results. According to the average ranking, the DESMA algorithm is in the leading position, and the *R*^*α*^ value is far lower than other algorithms, which shows that the DESMA algorithm has the best effect and the strongest stability. To sum up, the proposed DESMA algorithm has a positive effect. It not only helps the MA algorithm to jump out of the local optimum but also greatly improves the convergence speed and convergence accuracy.

The results of the *t*-test (significance level 0.05) of 28 functions are presented at the bottom of Table 3, in which "+" represents that an algorithm is better than the compared one, " = " indicates they have no differences, and "-" indicates the otherwise. From the results, we can see that our method outperforms the others significantly.

**Fig 2:**
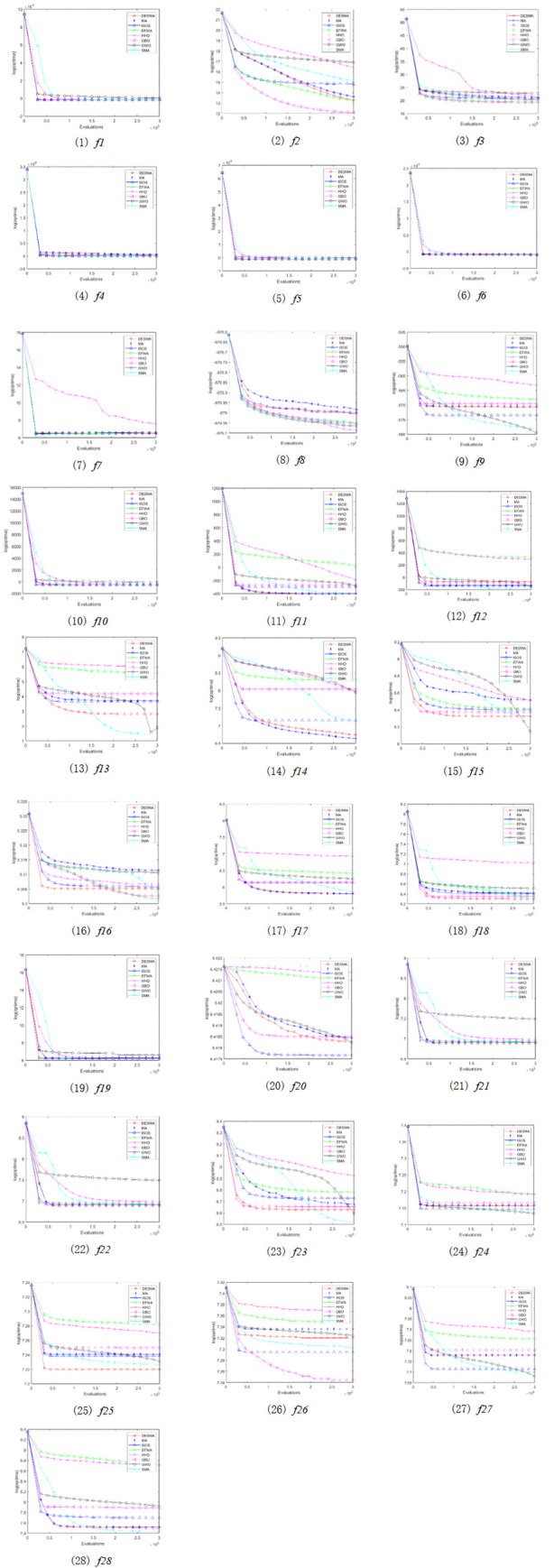
The average convergence curve of the algorithm under different calculation examples ((1)-(28) correspond to functions *f1*-*f28* respectively).

To verify the convergence speed and convergence accuracy of the DESMA algorithm, this paper designs the average convergence curve of the algorithm, as shown in Fig 2. It can be seen from Fig 2 that the eight algorithms have different evolutionary trends, and the DESMA algorithm is stronger than other algorithms in terms of convergence accuracy. On the functions *f*21, *f*22, and *f*28, the convergence speed of DESMA is slightly lower than that of EFWA, but its convergence accuracy is slightly stronger than that of MA and far stronger than ISOS, EFWA, HHO, GWO, GBO, and SMA. Moreover, on the functions *f*12, *f*18, *f*21, *f*22, *f*23, and *f*28, the DESMA algorithm is in the first position in terms of both the convergence speed and convergence accuracy. It is proved that adding a search radius near the global optimal solution in the DESMA algorithm can effectively prevent the algorithm from falling into the local optimal solution, find the global optimal solution more stably, and further improve the convergence performance of the algorithm.

**Fig 3:**
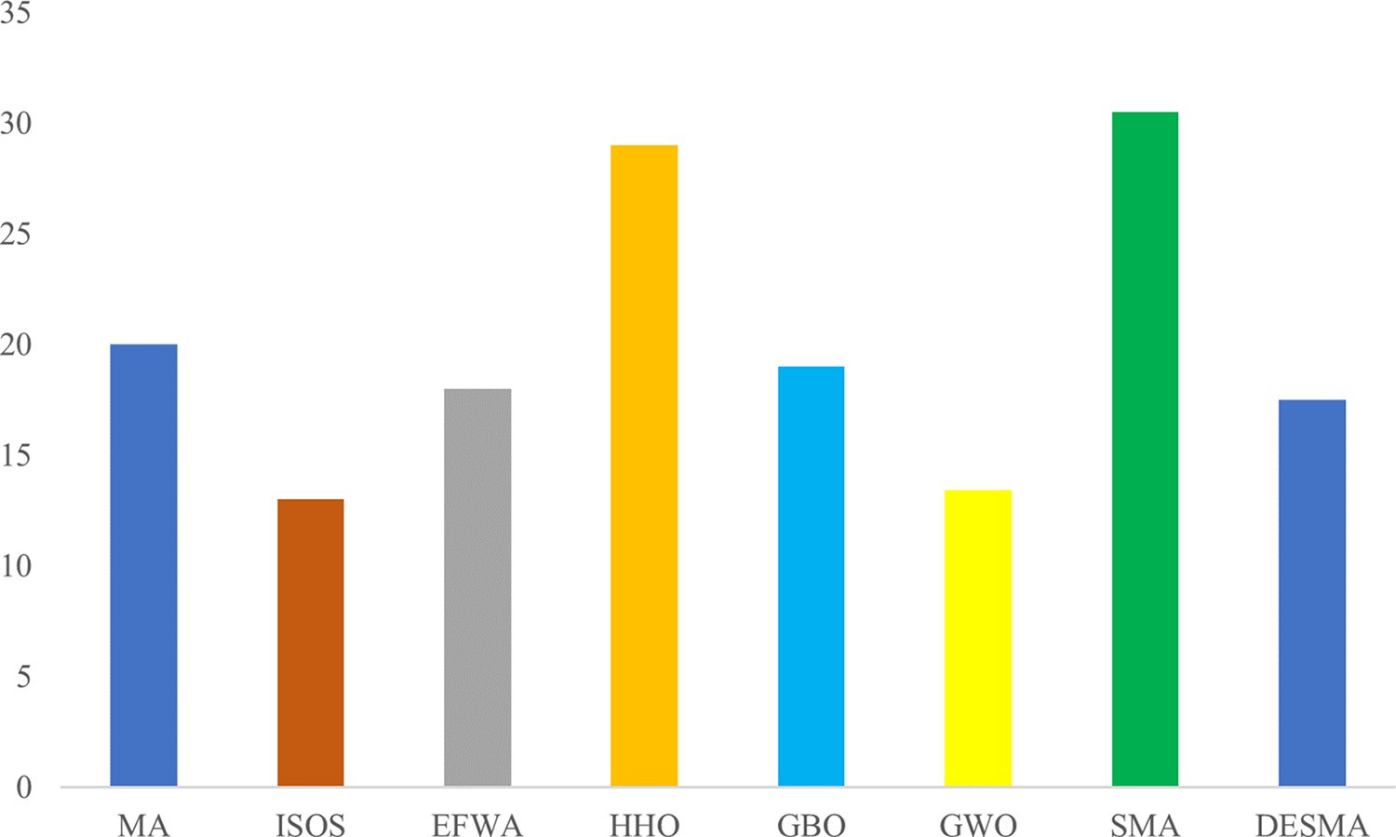
Comparison statistics of algorithm running time under different algorithm.

As can be seen from Fig 3, the ISOS and GWO algorithms have a shorter running time, followed by EFWA, DESMA, MA, HHO, GBO, and SMA are the worse among them in terms of running time. Although the DESMA algorithm isn’t the best among them, its optimization ability is better than the compared algorithms, and thus it can be considered acceptable.

## 5. Conclusion

To avoid falling into the local optimum, improve the searchability and convergence accuracy of MA. This work designs a dynamic elite strategy, thereby an improved MA is proposed. It first determines a specific space near the best mayfly in the current population and set the search radius dynamically. If the current global optimal solution is better than the previous generation global optimal solution, the search range of the elite mayfly will be expanded, otherwise, narrow the search range. Then, generating a certain number of elite mayflies within this range, selecting the elite mayfly with the best fitness value to replace the best mayflies in the current population if its fitness value is better than that of the current best one. This work conducts simulation experiments on the performance of the DESMA algorithm from various aspects and uses 28 benchmark test functions of benchmark to compare DESMA with MA, ISOS, EFWA, and HHO algorithms. The experimental results show that DESMA achieves better results on most functions, and the average ranking takes the place. At the same time, the convergence speed and convergence accuracy of the DESMA algorithm is greatly improved compared with its peers.

Even though the proposed algorithm has shown the superiority on 28 functions, it has the following limitations: 1) the enlargement and reduction factors in the proposed algorithm are set to fixed values, which cannot be adaptively adjusted for different functions during evolution; and 2) the proposed algorithm is not applied to real-world problems.

In the future, applying the proposed algorithm to optimize the real-world complex engineering problems [52–54] has become the key research direction for the next step. At the same time, the methods and related applications based on parameter adaptation are also key issues to be considered [55–58].

## Supporting information

S1 Data (RAR)
